# A multicomponent, high-intensity, patient-centered care intervention to optimize transitional care coordination for complex multimorbid people: a pre-post design

**DOI:** 10.3389/fmed.2025.1650973

**Published:** 2025-10-24

**Authors:** Tasmania del Pino-Sedeño, Beatriz González de León, Miguel García Hernández, Paula Coronil Olmedo, Yeiza Semiramis Reyes Melián, Vanesa Martínez Hernández, Estefanía García Bautista, Encarnación Barrios Arraez, Silvia Barreto Cruz, Alejandra Abrante-Luis, Miguel Angel García-Bello, Yadira González-Hernández, Juan Antonio López-Rodríguez, José Ramón Vázquez-Díaz

**Affiliations:** 1 Canary Islands Health Research Institute Foundation (FIISC), Tenerife, Spain; 2 Evaluation Unit (SESCS), Canary Islands Health Service (SCS), Tenerife, Spain; 3 Network for Research on Chronicity, Primary Care, and Health Promotion (RICAPPS), Tenerife, Spain; 4 Faculty of Health Sciences, Universidad Europea de Canarias, Tenerife, Spain; 5 Gerencia de Atención Primaria del Área de Salud de Tenerife, Tenerife, Spain; 6 Unidad Docente Multiprofesional de Atención Familiar y Comunitaria "La Laguna Tenerife Norte", Gerencia de Atención Primaria del Área de Salud de Tenerife, Tenerife, Spain; 7 Research Unit, Primary Health Care Management of Madrid, Madrid Health Service, Madrid, Spain; 8 Medical Specialties and Public Health Department, University Rey Juan Carlos, Madrid, Spain; 9 Primary Care Health Center General Ricardos, Madrid Health Service, Madrid, Spain

**Keywords:** multimorbidity, transitional care, patient-centered care, adherence, quality of life, treatment burden, integrated care, SPICA program

## Abstract

**Introduction:**

Complex multimorbid patients often experience uncoordinated care transitions, increasing the risk of poor adherence, fragmented care, and adverse outcomes. Multicomponent, patient-centered interventions may improve transitional care, but evidence remains limited and heterogeneous.

**Methods:**

This pre-post intervention study evaluated the impact of SPICA, a multicomponent, high-intensity, patient-centered transitional care program implemented in Tenerife, Spain. Eligible adult patients with multimorbidity and complexity were consecutively enrolled between September 2023 and June 2024. Primary outcome was adherence to pharmacological treatment (Morisky Medication Adherence Scale-4). Secondary outcomes included patient satisfaction (Baker’s Questionnaire), health-related quality of life (HRQoL, EQ-5D-5L), disease (Disease Burden Morbidity Assessment), and treatment burden (Treatment Burden Questionnaire). Outcomes were assessed at baseline and one-month post-discharge. Multivariate linear regression was used for the satisfaction outcome, and bivariate models were conducted to explore predictors of the remaining intervention outcomes. McNemar’s Chi-squared test was used to evaluate changes in adherence rates, and ANCOVA models for other outcomes measured at both pre- and post-intervention.

**Results:**

Among the 112 patients, adherence improved from 53.4 to 84.9% (*p* < 0.001). Satisfaction with care was high (median 71; IQR 67–81). Significant improvements were observed in HRQoL (mobility [*β* − 0.56], pain/discomfort [*β* − 0.55], anxiety/depression [*β* − 0.37], EQ-5D Index [*β* 0.14], EQ-VAS [*β* 7.08]), and treatment burden (*β* − 12.24). Baseline scores were the most consistent predictors of improvement; age, sex, and comorbidity were not significant factors.

**Discussion:**

A multicomponent, high-intensity, patient-centered intervention such as SPICA appears to be associated with improvements in adherence and health outcomes in complex multimorbid patients transitioning from hospital to primary care, and may also be linked to high levels of patient satisfaction. Effects were more pronounced in those with worse baseline scores, suggesting a positive impact among those most in need. Nevertheless, further studies with more robust methodological designs are required to confirm these associations.

## 1 Introduction

Multimorbidity, defined as the coexistence of two or more chronic health problems in the same individual (1), has become a predominant challenge for health systems worldwide. The global prevalence of multimorbidity has been estimated at 42.4% (2). In Europe, the prevalence of multimorbidity among adults over 50 years was 28.2% in men and 34.5% in women (3). This condition has important consequences on mortality, quality of life (4), and health costs (5). In addition, the construct of complexity is related to multimorbidity, although it is not a necessary or sufficient condition on its own. Chronic diseases coexist with social and environmental conditions that have an impact on self-care and hinder access to resources, worsening health outcomes and increasing hospital admission and readmission rates (6). From this perspective, complexity not only depends on health-related characteristics, but also on socioeconomic, cultural, environmental and care ones (7). Thus, some of the factors that have been identified in the literature as influencing complexity are polypharmacy (8, 9), functional or mobility limitations (10), difficulties in understanding (9, 10), cognitive impairment (11), limited access to resources (9, 12), as well as disease and treatment burden (10), among other factors. Complexity could be understood as a dynamic state in which personal, social and biological aspects of patients operate as factors that add difficulty (13). As such, complex multimorbid patients represent a vulnerable group within the population.

In this context, the transition of care from primary care to the hospital and subsequently from the hospital to primary care is a critical point, which is a concern for health systems around the world (14, 15). This point is frequently associated with uncoordinated continuity of care, errors in treatment and follow-up of care plans (16), and difficulties in follow-up, which increases the risk of hospital readmissions, low adherence to treatments and decreased patient satisfaction (17–19).

Although it is clear that therapeutic adherence is essential to achieve positive clinical results, especially after hospital discharge, recent studies show that up to 50% of patients with multimorbidity do not adequately comply with the prescribed treatments (20). This situation has been associated with various factors such as the complexity of the regimens, including treatment with multiple medications (polypharmacy) (21), the presence of adverse effects (22) and the lack of coordination and adequate follow-up (23).

To address these challenges, different types of interventions have been designed and implemented. Among them, multicomponent interventions, that is, those that combine strategies such as personalized health education, interprofessional coordination and the use of technologies for remote monitoring, have proven to be effective (10, 24). These interventions seek not only to improve adherence to treatment but also to reduce the burden perceived by the patient and increase their satisfaction with the care received (25). Evidence suggests that the effective integration of these strategies could facilitate safer and more efficient care transitions, reducing the gaps between levels of care. However, the heterogeneity in the design and implementation of these interventions makes it difficult to comparatively evaluate their effectiveness, highlighting the need for more robust and specific studies.

Among these interventions, the SPICA program (Subprograma de Integración y Coordinación Asistencial - Subprogram for Care Integration and Coordination) incorporates the components identified in the literature as essential to effective transitional care. It stands out as a multicomponent and high-intensity care initiative carried out at the Hospital Universitario de Canarias, Spain. This program is led by a multidisciplinary team, including family doctors, nurses, and social workers, who provide specialized care for complex multimorbid patients during care transitions. SPICA program aims to ensure social and family reintegration; enhance primary healthcare continuity for discharged patients; improve clinical outcomes through a structured, patient-centered approach (26); and incorporating and developing the core elements of the Chronic Care Model (27–30).

The present study evaluates the impact of a multicomponent, high-intensity, and patient-centeredness intervention (SPICA program) on complex multimorbid patients during the transition from primary care to hospital and subsequent return to primary care.

## 2 Materials and methods

### 2.1 Study design

A pre-post multicomponent, high-intensity, and patient-centered intervention study was conducted and reported in accordance with the Transparent Reporting of Evaluations with Nonrandomized Designs (TREND) statement (31).

### 2.2 Participants

Patients routinely accessed the SPICA program, where their eligibility for the study was verified based on specific selection criteria.

Patients are recruited and included in the SPICA program via two main pathways (26):

1. Hospital inpatient screening: The SPICA team’s professionals identify hospitalized patients as a high-risk population, particularly in terms of continuity of care after discharge, selecting those who may benefit most from specialized care.
2. Opportunistic recruitment: Patients can be referred by the service responsible for the hospital admission, primary care professionals, social workers, family members or through self-request.

For study inclusion, patients had to:

1. Be actively enrolled in the SPICA program.
2. Meet the following specific eligibility criteria, in line with those established by the SPICA program:
  - Adults aged 18 years and older.
  - Classified as complex patients due to multimorbidity, defined by:
    - ≥2 chronic health problems; and
    - Cognitive impairment (Pfeiffer Questionnaire > 4) (32); or
    - Dependency in activities of daily living (Katz Index of Independence in Activities of Daily Living [Katz ADL] > 1 or Lawton-Brody Instrumental ADL Scale < 6) (33, 34); or
    - Living alone; or
    - At least three of the following minor criteria:
      1. Age over 74
      2. Severe visual impairment
      3. Severe hearing impairment
      4. Malnutrition
      5. Poor self-perceived health
  - Provide informed consent to participate in the study.

### 2.3 Setting and recruitment

The study was conducted within the Primary Care Management of Tenerife under the Canary Islands Health Service (SCS), specifically at the Teaching Unit of Family and Community Care “La Laguna-Tenerife Norte,” located in Hospital Universitario de Canarias.

Recruitment was consecutive and conducted at the same hospital using the same mechanisms as the SPICA program. The enrolment in the SPICA program was a prerequisite for study inclusion. All patients who met both the SPICA program criteria (26) and the study’s eligibility requirements, and provided informed consent within the specified timeframe, were included. The recruitment period was between September 2023 and June 2024. The recruitment ended earlier than expected due to the closure of the program.

Accepted patients signed an informed consent form and completed the baseline questionnaires. In cases where the patient presented cognitive impairment, consent was provided by their legal representative.

### 2.4 Intervention

The SPICA program provided a multicomponent, high-intensity, and patient-centered care intervention aimed at ensuring continuity of care for hospitalized complex patients (26). It incorporated comprehensive biopsychosocial assessment, multidisciplinary coordination, and shared decision-making to improve patient outcomes. The intervention included:

1. A comprehensive biopsychosocial evaluation covering medical, functional, psychological, and contextual and social aspects.
2. The design and implementation of individualized care plans based on patient needs, preferences, and clinical evidence.
3. Regular case conferences with hospital and primary care professionals, occasionally including patients and their families.
4. Patient and caregiver support, with a focus on promoting self-care training, empowering the patient, providing emotional support, and enhancing the patient-clinician relationship.
5. A structured discharge plan to ensure proper transition to outpatient or home care.

The intervention was delivered face-to-face during hospital admission and continued after discharge through primary care follow-ups. It involved:

1. Direct patient-professional interactions (bedside visits, structured interviews, and shared decision-making sessions).
2. Care coordination meetings among hospital specialists, primary care professionals, and social workers.
3. Ongoing communication between SPICA professionals and primary care professionals to ensure follow-up and adherence to the care plan.

The intervention was performed by a multidisciplinary team composed of SPICA professionals, including family doctors and primary care nurses, in functional alliance with other medical specialists from both medical and surgical specialties, social workers, and nurses specialized in various areas depending on the case they attend to, as well as other healthcare and administrative professionals involved in patient care.

The intervention was delivered in two phases, beginning with an in-hospital phase that involved regular bedside visits and assessments throughout hospitalization, followed by a post-discharge phase focused on follow-up and coordination with primary care services, including communication with primary care professionals and adjustments to the care plan based on each patient’s needs, ensuring continuity of care.

In order to enhance adherence to the care plan and ensure treatment continuity, the intervention involved frequent patient-caregiver interactions, personalized self-care education, family involvement in decision-making, and close follow-up with primary care professionals. Further details on the design, implementation, and evaluation of the SPICA intervention are available in García Hernández et al. (26).

### 2.5 Objectives

The objectives of the present study are:

1. To evaluate the impact of SPICA intervention—a multicomponent, high-intensity, and patient-centered care approach designed to ensure continuity of care for hospitalized complex multimorbid patients—by assessing its effects on adherence, patient satisfaction, and health outcomes, including health-related quality of life (HRQoL), disease burden, and treatment burden.
2. To identify protective and risk factors influencing adherence, patient satisfaction, and HRQoL in this population.

The hypothesis is that improvements in adherence, satisfaction, and HRQoL levels following SPICA intervention are influenced by patients’ social, clinical, and personal factors.

### 2.6 Outcomes

#### 2.6.1 Primary outcome

Patient adherence to pharmacological treatment assessed by the Spanish validated version of the Morisky Medication Adherence Scale-4 (MMAS-4™). This self-reported measure consists of four dichotomous (“yes” or “no”) questions designed to identify barriers to proper therapeutic adherence across a variety of chronic medical conditions. The scale has been shown to have moderate reliability (*α* = 0.62) (35–38). Permission to use the MMAS-4™ was obtained through a formal license agreement. All conditions for the authorized use of the instrument were fulfilled in accordance with the licensing agreement.

For the analyses, adherence levels were categorized into two groups: ‘adherent’ (all responses indicate adherence) versus ‘non-adherent’ (at least one response does not reflect adherence). Patient classification as adherent or non-adherent was determined based on their adherence to all medications within their treatment regimen. Adherence was assessed at baseline, and one-month post-discharge.

#### 2.6.2 Secondary outcomes

Satisfaction with general practice consultations was measured one-month post-discharge using the Spanish version of Baker’s questionnaire (39).

Baker’s questionnaire is a valid and reliable self-reporting scale (39), consisting of 18 items, each answered on a 5-point Likert scale (ranging from ‘totally disagree’ to ‘totally agree’). It consists of three dimensions: care provided by the professional, time spent during the consultation, and the depth of the relationship with the professional. This scale helps identify areas of patient-perceived strengths and weaknesses in general practice.

Other secondary outcomes were assessed at baseline and one-month post-discharge, including: HRQoL, measured with the Spanish version of EuroQol-5-Dimension-5-Level (EQ-5D-5L); disease burden, assessed using the Spanish version of Disease Burden Morbidity Assessment (DBMA) (40); Treatment burden and its impact on patient well-being, evaluated through the Spanish version of Treatment Burden Questionnaire (TBQ) (41).

EQ-5D-5L is a reliable and validate self-administered instrument, divided into two sections (42): the descriptive system and the Visual Analogue Scale (VAS). The first section assesses an individual’s current health status across five dimensions: mobility, self-care, usual activities, pain/discomfort, and anxiety/depression. Each dimension includes five response levels, representing increasing severity: no problems (1), slight problems (2), moderate problems (3), severe problems (4), and extreme problems or inability (5). Each respondent selects the level that best reflects their status for each of the five dimensions. The second section asks respondents to rate their current health status using a 20-cm vertical scale, resembling a thermometer. The top of the scale, marked as ‘the best health state you can imagine,’ is assigned a value of 100, while the bottom, labeled ‘the worst health state you can imagine,’ is assigned a value of 0. Additionally, the five-digit health states from the descriptive system can be converted into a single utility index score (EQ-5D Index) using country-specific value sets. This index, which typically ranges from values below 0 (indicating health states perceived as worse than death) to 1 (full health), reflects societal preferences for different health states (43).

DBMA has demonstrated satisfactory feasibility and acceptability, with a Cronbach’s alpha of 0.72 (40). This self-reported instrument includes 21 common medical conditions. Patients first indicate whether they have any of the listed conditions and, if applicable, rate the extent to which each condition limits their daily activities using a Likert scale ranging from ‘none’ (1) to ‘a lot’ (5). The total score is calculated as the sum of limitation scores across all conditions, with higher scores reflecting greater limitations in activities of daily living.

TBQ is a self-administered reliable instrument (*α* > 0.8) (41). The scale has 16 items, each rated on a Likert scale from 0 (‘no effort’) to 10 (‘extreme effort’). The scores for all items are summed to generate a total score ranging from 0 to 160, with higher scores indicating a greater treatment burden.

#### 2.6.3 Additional sociodemographic and clinical variables

In addition, the following measures were collected at baseline from the patients: Functional status, specifically the ability to perform activities of daily living independently, was assessed using the Spanish version of Katz ADL Index (33, 34) and Lawton-Brody Instrumental ADL Scale; comorbid disease burden, used to predict the risk of one-year mortality in hospitalized patients based on the presence of specific chronic health conditions, was assessed using the Charlson Comorbidity Index (44, 45); cognitive function, specifically that indicative of cognitive impairment, was evaluated using the Spanish version of Pfeiffer test (32, 46); and sociodemographic data (age, sex, education level, marital status, number of children, family type, and cohabitants) were collected through an *ad hoc* questionnaire.

Finally, clinical data, including number of prescribed drugs—categorized based on the presence or absence of polypharmacy, defined as the routine use of five or more medications (47)—number of chronic health problems and medical specialties involved in patient care, were collected from the electronic health records and defined based on the information available at the time of the patient’s hospitalization.

#### 2.6.4 Data collection

The data was obtained from two different sources: the patients themselves, as well as information obtained from the electronic health records from primary care (Drago AP) and specialized care (SAP), and continuous electronic prescription of the Canary Islands Health Service.

All questionnaires, including those on sociodemographic characteristics, MMAS-4™, Baker’s questionnaire, EQ-5D-5L, DBMA, TBQ, Katz ADL Index, Charlson Comorbidity Index, and the Pfeiffer test, were administered face-to-face by a SPICA professional during the patient’s hospital admission interview. Follow-up assessments were conducted via a telephone interview, for measures also collected post-intervention, between the SPICA family doctor/nurse and the patient. The clinical data of all patients were collected from electronic health records by SPICA professionals.

All the information was stored in a protected Excel document that met the required confidentiality criteria.

### 2.7 Sample size

A two-tailed test at a 5% significance level and 80% power, accounting for a 20% loss to follow-up, determined that 264 patients were required to detect a 12.5% difference in treatment adherence, using the MMAS-4™, according to previous literature (48).

### 2.8 Assignment method

Since this study followed a pre-post intervention design with a single-arm approach, no randomization or group assignment was performed. Instead, all eligible participants were consecutively included in the study upon meeting the inclusion criteria.

### 2.9 Blinding

No blinding was employed in this study, as it followed an open-label pre-post intervention design.

### 2.10 Unit of analysis

The individual was the unit of analysis in this single-arm study.

### 2.11 Statistical analyses

Continuous variables were summarized using means and standard deviations (SD) or medians and interquartile ranges (IQR), depending on their distribution. Categorical variables were presented as frequencies and percentages.

Differential adherence rates for the main variable were analyzed with McNemar’s Chi-squared test with continuity correction. Changes in each of the five EQ-5D-5L domains (mobility, self-care, usual activities, pain/discomfort, and anxiety/depression) were assessed using the Wilcoxon signed-rank test.

Bivariate regression analyses were conducted to explore associations between sociodemographic factors (e.g., sex and age) and clinical characteristics (e.g., number of chronic health problems, number of prescribed drugs, number of dependencies, Katz Index score, and number of medical specialties involved), and the change scores of the outcome measures (i.e., the difference between post- and pre- intervention scores) for adherence, health-related quality of life (HRQoL), disease burden, and treatment burden. Univariate linear regression analyses were performed for satisfaction, which was assessed only at post-intervention. Variables yielding a *p*-value ≤ 0.10 in the previous analyses were considered for inclusion in subsequent models as covariates. If the variables were not significant, the models were adjusted for age, sex, and baseline outcome measure score.

Finally, for outcomes with both pre- and post-intervention measurements (e.g., adherence, HRQoL, disease burden, and treatment burden), analyses of covariance (ANCOVA) models were applied to assess changes over time. A linear link function was used for continuous dependent variables. In contrast, satisfaction—assessed only once at the post-intervention time point—was analyzed using multivariate linear regression.

All analyses followed an intention-to-treat approach. Statistical analyses were conducted using R version 4.3. (49).

### 2.12 Ethical consideration

The study was conducted according to the guidelines of the Declaration of Helsinki, applicable local legislation and institutional requirements, and was approved by the Clinical Research Ethics Committee with Medicines of Hospital Universitario de Canarias (CHUNSC_2023_97 [PIFIISC22/25]).

## 3 Results

### 3.1 Participant flow and recruitment

**Figure 1:**
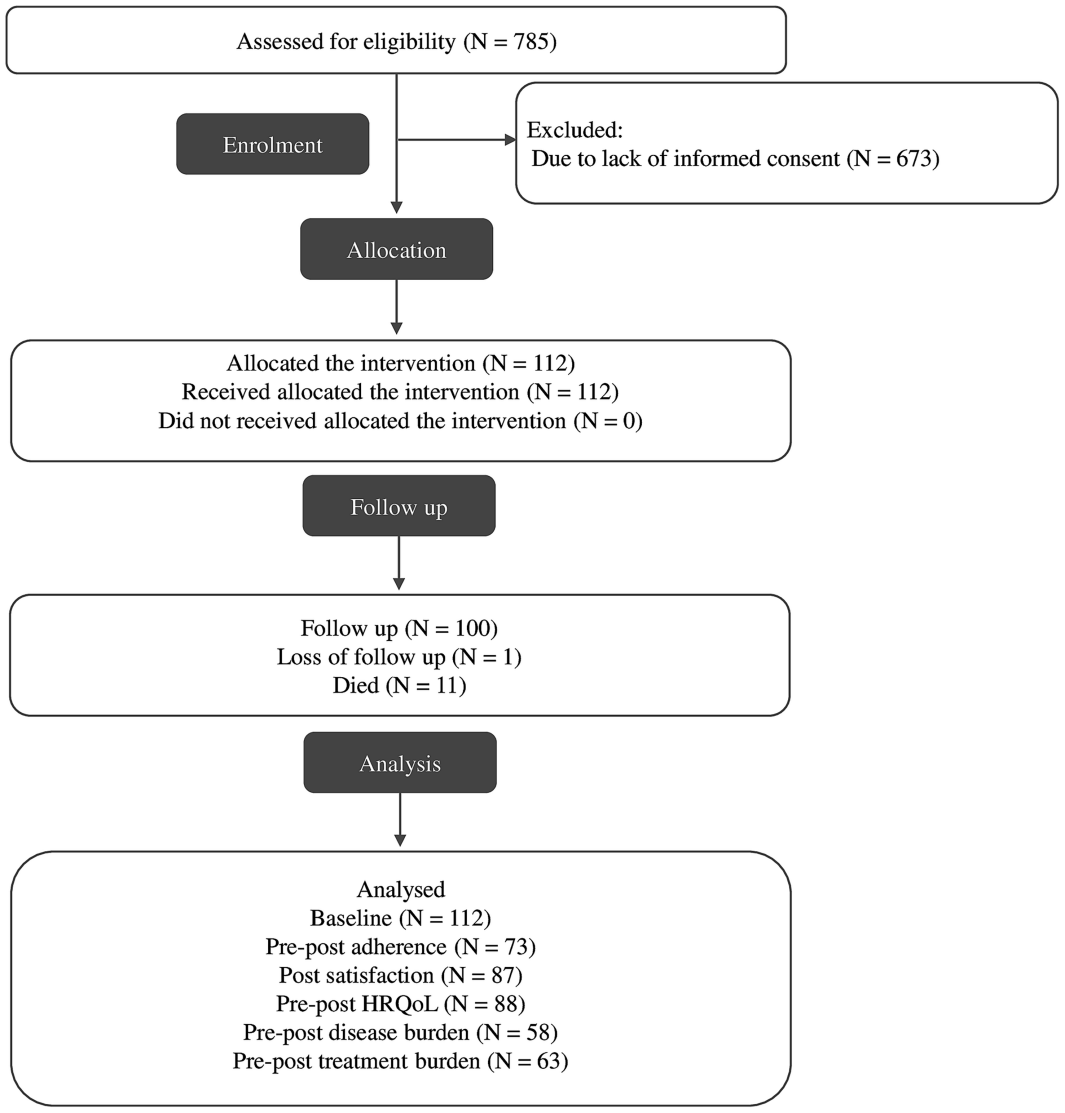
Flowchart of selection process.

Out of the 785 patients attended to in the SPICA program between September 2023 and July 2024, 112 individuals were enrolled in the study (see Figure 1). Eleven participants (9.82%) died before the one-month follow-up, and 100 participants completed the intervention and had evaluable pre- and post-intervention data. These were included in the final per-protocol analysis. Participants who withdrew consent, were lost to follow-up, or had incomplete data on primary outcomes were excluded from the analysis. No imputation methods were applied for missing data.

The recruitment process was prematurely halted due to the external discontinuation of the SPICA program, a system-level decision unrelated to the study protocol.

### 3.2 Baseline sample characteristics

At baseline, the mean age was 76.13 years (SD ± 10.73), ranging from 40 to 94 years. Approximately half of the participants were men, and the other half were women. Only about half were married or in a relationship, although more than 80% lived with a partner, family, or friends. On average, participants had 9.65 (SD ± 5.37) health conditions and over 80% of participants presented with polypharmacy.

A total of 19 participants (16.97%) had moderate to severe cognitive impairment (Pfeiffer Questionnaire > 4), while 74 (66.07%) had significant dependency in ADL (Katz ADL Index score ≠ A). Regarding healthcare resource utilization, participants had an average of one hospital admission in the previous year (SD ± 0.15) and 20.64 primary care visits (SD ± 1.73). Table 1 summarizes the participants’ baseline characteristics.

**Table 1:** Baseline demographic and clinical characteristics of patients.

Variables	Total (*N* = 112)
Age, *N* (%)	
<54 yrs.	3 (2.68)
55–64 yrs.	13 (11.61)
65–79 yrs.	49 (43.75)
>80 yrs.	47 (41.96)
Sex, *N* (%)	
Female	54 (48.21)
Male	58 (51.79)
Education level, *N* (%)	
No formal education	31 (27.68)
Primary education	58 (51.79)
Secondary education	12 (10.71)
University education	10 (8.93)
Marital status, *N* (%)	
Single	10 (8.93)
Married/partnered	53 (47.32)
Separated/divorced	10 (8.93)
Widowed	37 (33.04)
Other	1 (0.89)
Children, *N* (%)	
Yes	100 (89.29)
No	11 (9.82)
Type of family, *N* (%)	
Nuclear	73 (65.18)
Binuclear	1 (0.89)
Single-parent	2 (1.79)
Extended	7 (6.25)
Family equivalent	9 (8.04)
Lives alone	20 (17.86)
Cohabitants, *N* (%)	
Alone	28 (25.00)
Partner	32 (28.57)
Family/friends	52 (46.43)
Number of prescribed drugs, M (SD)	9.67 (5.12)
No polypharmacy (0–4), *N* (%)	20 (17.86)
Polypharmacy (≥5), *N* (%)	92 (82.14)
Number of Chronic Health Problems, M (SD)	9.65 (5.37)
Charlson Comorbidity Index, M (SD)	6.66 (2.50)
Katz ADL Index, M (SD)	3.28 (2.39)
Dependent on at least in one activity, *N* (%)	74 (66.07)
Independent for all activities, *N* (%)	38 (33.93)
Lawton-Brody instrumental ADL scale, *N* (%)	
Total dependence (0–1)	24 (21.43)
Severe dependence (2–3)	10 (8.93)
Moderate dependence (4–5)	9 (8.04)
Mild dependence (6–7)	13 (11.61)
Independent (8)	55 (49.11)
Missing	1 (0.89)
Pfeiffer questionnaire, *N* (%)	
Normal (0–2)	78 (69.64)
Mild impairment (3–4)	13 (11.61)
Moderate impairment (5–7)	10 (8.93)
Severe impairment (8–10)	9 (8.04)
Missing	2 (1.79)
Psychoaffective problem, *N* (%)	
Yes	50 (44.64)

ADL, Activities of Daily Living; IQR, Interquartile range; M, mean; N, number of patients; SD, standard deviation; yrs., Years.

### 3.3 Outcomes and estimation

#### 3.3.1 Primary outcome: adherence

Among the 73 patients evaluated at both time points, adherence improved significantly from 39 patients (53.42%) at baseline to 62 patients (84.93%) post-intervention (*p* < 0.001). Of the 39 patients initially classified as adherent, 34 remained adherent, while five became non-adherent. Conversely, among the 34 patients who were non-adherent at baseline, 28 became adherent, while six remained non-adherent.

Table 2 shows the bivariate regression model examining the association between patients’ change in adherence score and baseline sociodemographic and clinical characteristics. No association was found between differences in adherence score and any of the variables analyzed, including sex, age, Charlson Comorbidity Index, number of chronic health problems, number of prescribed drugs, functional status (Katz ADL Index), or the number of medical specialties involved.

**Table 2:** Association between baseline sociodemographic and clinical characteristics and patients’ change in adherence score (bivariate regression).

Predictors	*β*	95% CI	*p*
Sex (Women; Reference: Men)	−0.03	−0.57, 0.50	0.90
Age (years)	−0.02	−0.04, 0.00	0.12
Charlson Comorbidity Index	−0.05	−0.15, 0.06	0.35
Number of Chronic Health Problems	0.01	−0.04, 0.06	0.82
Number of prescribed drugs	−0.03	−0.07, 0.02	0.31
Katz ADL Index	0.09	−0.02, 0.21	0.12
Medical specialties involved	0.01	−0.10, 0.13	0.83

ADL, Activities of Daily Living; *β*, Regression coefficient; CI, Confidence Interval; *p*, *p*-value.

The SPICA intervention resulted in a statistically significant improvement in medication adherence, as evidenced by a reduction in MMAS-4™ scores from pre- to post-intervention. The regression model confirmed that higher baseline MMAS-4™ scores were predictive of greater improvement post-intervention (*β* − 0.95). Notably, neither age nor sex were significant predictors, suggesting that the observed improvements in adherence were not associated with demographic factors (Table 3).

**Table 3:** Change scores and associated predictors for adherence, HRQoL, and disease and treatment burden scores.

Outcomes	Crude change (Post vs Pre)			Adjusted models for change scores										
				Intercept		Baseline score		Age		Sex		Other variables^3^		Model fit *R*^2^; adj *R*^2^
	Pre	Post	*p*	*β*	*p*	*β*	*p*	*β*	*p*	*β*	*p*	*β*	*p*	
Non-Adherence (MMAS-4™ Score), M (SD)	0.8 (1.0)	0.2 (1.0)	**<0.001** ^**1**^	**−0.59**	**<0.001**	**−0.95**	**<0.001**	−0.01	0.30	0.14	0.39			0.70; 0.69
EQ-5D-5L Mobility, Median (IQR)	3.0 (2.0–4.0)	2.5 (2.0–4.0)	**<0.001** ^**1**^	**−0.56**	**<0.001**	**−0.46**	**<0.001**	0.01	0.59	0.05	0.83			0.23; 0.21
EQ-5D-5L Self-care, Median (IQR)	3.0 (2.0–4.0)	3.0 (1.0–5.0)	0.59^1^	−0.15	0.52	**−0.50**	**<0.001**	0.01	0.38	0.04	0.89	**0.21**	**<0.01**	0.22; 0.18
EQ-5D-5L Usual activities, Median (IQR)	3.5 (2.0–5.0)	3.0 (1.0–5.0)	0.51^1^	−0.16	0.50	**−0.49**	**<0.001**	0.03	0.05	0.05	0.88	0.05	0.09	0.29; 0.25
EQ-5D-5L Pain/ Discomfort, Median (IQR)	2.0 (1.0–3.0)	1.0 (1.0–2.0)	**0.009** ^**1**^	**−0.55**	**<0.01**	**−0.80**	**<0.001**	−0.03	0.80	0.03	0.92			0.46; 0.43
EQ-5D-5L Anxiety/ Depression, Median (IQR)	2.0 (1.0–3.0)	1.0 (1.0–3.0)	**0.02** ^**1**^	**−0.37**	**<0.05**	**−0.84**	**<0.001**	−0.01	0.44	−0.07	0.78			0.51; 0.49
EQ-VAS, M (SD)	58.34 (27.68)	63.96 (22.95)	0.08^2^	7.08	0.05	**0.83**	**<0.001**	0.17	0.44	−2.25	0.66			0.58; 0.56
EQ-5D Index, M (SD)	0.47 (0.32)	0.58 (0.29)	**0.001** ^**2**^	**0.14**	**<0.001**	**−0.63**	**<0.001**	−0.00	0.83	−0.03	0.59			0.37; 0.35
Disease burden (IQR)	8.0 (5.0–13.0)	6.0 (3.0–9.75)	**0.007** ^**1**^	−1.27	0.21	**−0.68**	**<0.001**	−0.04	0.48	−2.19	0.12			0.67; 0.64
Treatment burden (IQR)	21.0 (6.0–48.5)	9.0 (0.0–21.0)	**<0.001** ^**1**^	**−12.24**	**<0.001**	**−0.79**	**<0.001**	0.09	0.42	**−10.92**	**<0.001**			0.84; 0.83

^1^Wilcoxon test; ^2^T-test; ^3^Katz ADL Index (for EQ-5D-5L Self-care) and Prescribed drugs (for EQ-5D-5L Usual activities); Adjusted *R*^2^ = Adjusted coefficient of determination (accounts for the number of predictors in the model); *β* = Regression coefficient; IQR = Interquartile range; M = Mean; *p*: *p*-value; Pre: Pre-intervention; Post: Post-intervention; *R*^2^ = Coefficient of determination; SD = standard deviation; SE = Standard error.

The quantitative variables have been included in the models centered on the mean.

The numbers in bold represent statistically significant differences.

#### 3.3.2 Secondary outcomes

##### 3.3.2.1 Satisfaction

Among participants (*N* = 87), the satisfaction score with general practice consultations had a median of 71 (IQR 67–81). Regression analyses revealed no statistically significant associations between post-intervention satisfaction scores and any of the sociodemographic or clinical variables analyzed, indicating a relatively uniform perception of satisfaction across demographic and clinical subgroups (Table 4).

**Table 4:** Univariate and multivariate linear regression models predicting satisfaction.

Predictors	Univariate			Multivariate		
	*β*	SE	*p*	*β*	SE	*p*
Sex (Women; Reference: Men)	1.76	2.31	0.76	0.62	2.43	0.80
Age	0.11	0.10	0.27	0.11	0.11	0.32
Charlson Comorbidity Index	−0.55	0.46	0.23	–	–	–
Number of Chronic Health Problems	−0.41	0.20	0.046	−0.41	0.21	0.051
Number of prescribed drugs	−0.18	0.22	0.41	–	–	–
Katz ADL Index	−0.86	0.49	0.08	−0.85	0.50	0.09
Medical specialties involved	−0.23	0.52	0.66	–	–	–

ADL, Activities of Daily Living; *β*, Regression coefficient; Katz Index, functional status scale assessing independence in basic ADLs; *p*, *p*-value; SE, Standard error.

The quantitative variables have been included in the models centered on the mean.

##### 3.3.2.2 HRQoL

Among the 88 patients evaluated at both time points, significant improvements were observed in three EQ-5D-5L dimensions: mobility (*p* < 0.001), pain/discomfort (*p* = 0.009), and anxiety/depression (*p* = 0.017). For mobility, the proportion of patients reporting “no problems” increased from 11 (9.82%) at baseline to 18 (16.07%) post-intervention, while those reporting “unable/extreme” problems decreased from 21 (18.75%) to 9 (8.04%). Similarly, for pain/discomfort, 42 patients (37.50%) initially reported “no problems,” increasing to 53 (47.32%) after the intervention. In anxiety/depression, 47 patients (41.96%) reported “no problems” at baseline, rising to 53 (47.32%) post-intervention, with a reduction in those reporting severe or extreme issues. In contrast, no significant changes were observed in the self-care and usual activities dimensions, with the distribution of responses remaining relatively stable (Table 5).

**Table 5:** Frequencies (%) and patient’s change in severity levels of EQ-5D-5L domains.

EQ-5D-5L Dimension/Scores	No problems		Slight problems		Moderate problems		Severe problems		Unable/extreme		*p*
	Pre (*n* = 109)	Post (*n* = 88)	Pre (*n* = 109)	Post (*n* = 88)	Pre (*n* = 109)	Post (*n* = 88)	Pre (*n* = 109)	Post (*n* = 88)	Pre (*n* = 109)	Post (*n* = 88)	
Mobility	11 (9.82)	18 (16.07)	25 (22.32)	26 (23.21)	27 (24.11)	21 (18.75)	25 (22.32)	14 (12.50)	21 (18.75)	9 (8.04)	<0.001^1^
Self-care	22 (19.64)	32 (28.57)	24 (21.43)	8 (7.14)	23 (20.54)	10 (8.93)	14 (12.50)	12 (10.71)	26 (23.21)	26 (23.21)	0.59^1^
Usual activities	20 (17.86)	23 (20.54)	15 (13.39)	12 (10.71)	19 (16.96)	10 (8.93)	13 (11.61)	7 (6.25)	42 (37.50)	36 (32.14)	0.50^1^
Pain/discomfort	42 (37.50)	53 (47.32)	23 (20.54)	14 (12.50)	20 (17.86)	11 (9.82)	19 (16.96)	7 (6.25)	5 (4.46)	3 (2.68)	0.009^1^
Anxiety/depression	47 (41.96)	53 (47.32)	21 (18.75)	11 (9.82)	22 (19.64)	17 (15.18)	11 (9.82)	3 (2.68)	8 (7.14)	4 (3.57)	0.017^1^

^1^Wilcoxon test; EQ-5D-5L, EuroQol 5-Dimension 5-Level scale; *p*, *p*-value.

Table 6 presents the results of the bivariate regression model examining the associations between patients’ changes in EQ-5D-5L domain scores and baseline sociodemographic and clinical characteristics. No statistically significant associations were found for sex, age, Charlson Comorbidity Index, number of chronic health problems, poorer functional status (measured by the Katz Index), or number of medical specialties involved. However, a higher number of prescribed drugs was marginally significantly associated with greater limitations in performing usual activities (*β* = 0.08, *p* = 0.02). Similarly, although age was not significantly related to HRQoL domains, a borderline relationship was observed between older age and increased difficulty in usual activities (*p* = 0.07).

**Table 6:** Association between baseline sociodemographic and clinical characteristics changes in EQ-5D-5L domains scores (bivariate regression).

Predictors	Mobility			Self-care			Usual activities			Pain/discomfort			Anxiety/depression			EQ-VAS			EQ-5D Index		
	*β*	95% CI	*p*	*β*	95% CI	*p*	*β*	95% CI	*p*	*β*	95% CI	*p*	*β*	95% CI	*p*	*β*	95% CI	*p*	*β*	95% CI	*p*
Sex (Women; Reference: Men)	0.07	−0.45, 0.59	0.78	0.21	−0.45, 0.88	0.53	0.24	−0.48, 0.96	0.51	−0.14	−0.78, 0.50	0.66	−0.00	−0.68, 0.67	0.99	8.93	−3.5, 21	0.16	−0.03	−0.17, 0.11	0.69
Age	0.00	−0.02, 0.03	0.87	0.01	−0.02, 0.04	0.65	0.03	0.00, 0.06	0.07	−0.01	−0.03, 0.02	0.71	−0.00	−0.03, 0.03	0.88	−0.08	−0.66, 0.48	0.76	0.00	−0.01, 0.01	0.97
Charlson Comorbidity Index	0.06	−0.05, 0.16	0.27	−0.08	−0.21, 0.05	0.23	0.01	−0.13, 0.16	0.87	0.09	−0.04, 0.22	0.19	−0.02	−0.16, 0.12	0.76	−0.57	−3.1, 1.9	0.65	−0.00	−0.03, 0.03	0.98
Number of Chronic Health Problems	0.03	−0.02, 0.08	0.21	0.01	−0.06, 0.07	0.87	0.03	−0.04, 0.10	0.38	−0.01	−0.07, 0.05	0.75	−0.03	−0.10, 0.03	0.30	0.17	−1.1, 1.4	0.79	−0.00	−0.01, 0.01	0.78
Number of prescribed drugs	0.01	−0.04, 0.06	0.68	0.03	−0.03, 0.09	0.33	0.08	0.02, 0.15	0.02	0.00	−0.06, 0.06	0.91	0.00	−0.06, 0.06	0.97	0.25	−0.93, 1.4	0.68	−0.01	−0.02, 0.01	0.33
Katz ADL Index	−0.03	−0.14, 0.09	0.65	0.12	−0.02, 0.26	0.10	−0.01	−0.16, 0.15	0.95	0.01	−0.13, 0.15	0.91	−0.00	−0.15, 0.14	0.99	−1.90	−4.5, 0.75	0.16	−0.00	−0.03, 0.03	0.76
Medical specialties involved	−0.04	−0.15, 0.08	0.53	−0.02	−0.17, 0.12	0.76	0.10	−0.05, 0.26	0.19	−0.05	−0.19, 0.09	0.46	−0.05	−0.20, 0.10	0.49	1.59	−1.2, 4.4	0.26	0.01	−0.02, 0.04	0.52

ADL, Activities of Daily Living; *β*, Regression coefficient; EQ-5D-5L, EuroQol 5-Dimension 5-Level scale; EQ-VAS, EuroQol Visual Analogue Scale (self-rated health from 0 = worst to 100 = best imaginable); Katz Index, functional status scale assessing independence in basic ADLs; *p*, *p*-value.

The quantitative variables have been included in the models centered on the mean.

The SPICA intervention was associated with statistically significant improvements in the mobility, pain/discomfort, and anxiety/depression dimensions of HRQoL. Additionally, improvements were observed in both overall health status (EQ-5D Index) and self-perceived health status (EQ-VAS). The regression model predicting changes in outcomes identified baseline scores as the strongest and most consistent predictors of improvement across most domains. Specifically, participants with worse initial scores experienced more pronounced improvements, particularly in the domains of mobility, pain/discomfort, and anxiety/depression, as well as in the overall EQ-5D score. Neither age nor sex emerged as significant predictors in most models, suggesting that the observed improvements were broadly consistent across demographic groups (Table 3).

##### 3.3.2.3 Disease and treatment burden

As shown in Table 7, neither sex, age, Charlson Comorbidity Index, number of chronic health problems, number of prescribed drugs, Katz Index, nor the number of medical specialties involved were significantly associated with either changes in disease burden or treatment burden.

**Table 7:** Association between baseline sociodemographic and clinical characteristics and pre-post changes in disease/treatment burden (bivariate regression).

Predictors	Disease burden			Treatment burden		
	*β*	95% CI	*p*	*β*	95% CI	*p*
Sex (Women; Reference: Men)	−3.67	−7.80, 0.45	0.08	−11.62	−24, 0.63	0.06
Age	−0.09	−0.26, 0.08	0.29	0.37	−0.14, 0.89	0.15
Charlson Comorbidity Index	0.48	−0.32, 1.3	0.24	1.80	−0.66, 4.3	0.15
Number of Chronic Health Problems	0.08	−0.33, 0.49	0.70	−0.12	−1.2, 1.0	0.84
Number of prescribed drugs	−0.17	−0.55, 0.22	0.39	−0.09	−1.3, 1.1	0.89
Katz ADL Index	−0.29	−1.2, 0.65	0.55	1.33	−1.5, 4.1	0.35
Medical specialties involved	−0.08	−1.0, 0.84	0.86	−0.13	−2.9, 2.7	0.93

ADL, Activities of Daily Living; *β*, Regression coefficient; CI, Confidence Interval; Katz Index, functional status scale assessing independence in basic ADLs; *p*, *p*-value.

The quantitative variables have been included in the models centered on the mean.

Both disease burden and treatment burden showed statistically significant improvements between pre- and post-intervention scores (Table 3).

The improvement in disease burden score was no longer statistically significant in the multivariate model (*p* = 0.21), whereas treatment burden continued to show a significant reduction. The multivariate regression model confirmed that these improvements were primarily predicted by participants’ baseline burden levels—those with higher initial scores experienced the greatest benefit. Although age was not significantly associated with changes, sex was a significant predictor of treatment burden improvement, with women showing greater reductions in burden compared to men.

## 4 Discussion

The main objective of this pre-post study was to assess the effects of the SPICA intervention on patients with complex multimorbidity, focusing on adherence, patient satisfaction, and health outcomes, such as HRQoL, disease burden, and treatment burden. Moreover, the study aimed to explore which patient characteristics were associated with greater response to the intervention in terms of satisfaction, adherence, HRQoL, and perceived burden.

The results suggest a potential improvement in medication adherence post-intervention. This is consistent with previous literature (50, 51). Notably, the patients who benefited the most were those with lower baseline adherence levels, highlighting the intervention could be particular useful in individuals with greater intervention needs in this regard. Furthermore, no significant associations were found between adherence outcomes and sociodemographic variables such as age or sex, suggesting that the observed improvements were consistent across demographic groups.

Previous studies have shown that patient satisfaction with transitional care interventions is associated with improved medication adherence, especially when these interventions include educational components, structured follow-up, and ongoing support (52). In our study, we found a high level of overall satisfaction, compared to other studies (53, 54), regardless of the patients’ demographic and clinical characteristics. These beneficial results on satisfaction in the care transition were expected, taking into account previous studies (55, 56). Several factors have been identified that potentially influence satisfaction in these situations, such as care coordination and continuity (55, 57), self-management education and ongoing support (55, 58), improved quality of life (57), multidisciplinary interventions (57, 59), and structured follow-up after discharge (60). In fact, single-component interventions are generally ineffective for patients with multimorbidity, and an integrated, multifaceted approach is recommended to optimize the care of these patients (60–62). In the case of the SPICA intervention, all of these factors are combined.

As we have already seen, HRQoL is a factor that directly influences patient satisfaction. In this study, HRQoL scores indicated that the SPICA intervention could have produced significant improvements in the mobility, pain/discomfort, and anxiety/depression domains. Overall health status also improved, and a positive change was observed in self-perceived health status. Specifically, those with more severe baseline problems—both in individual domains and in overall HRQoL—showed the greatest improvements. Once again, these results are consistent with previous literature. Transitional care interventions, especially those that are multidisciplinary and complex, have been shown to have a positive impact on the quality of life of these patients (57, 63). However, these results should be interpreted with caution, as baseline HRQoL and other clinical variables may have been temporarily affected by the recent hospitalization. In this context, part of the observed improvement could reflect a natural post-acute recovery process, rather than the sole effect of the intervention.

Regarding disease and treatment burden, both domains showed significant improvements following the intervention, which is consistent with previous literature (50). These improvements were primarily influenced by baseline burden levels, with participants experiencing higher initial burden reporting greater post-intervention reductions. Additionally, sex appeared to play a role, as women demonstrated more pronounced improvements in treatment burden.

The hypothesis that improvements in adherence, satisfaction, and HRQoL levels following the SPICA intervention would be influenced by patients’ social, clinical, and personal factors was only partially confirmed. Although improvements were consistently observed, most outcomes were not significantly associated with variables such as sex, age, or number of health problems. In fact, patient-centered interventions were found to improve discharge readiness, quality of transition, and HRQoL in both women and men, with no significant gender differences in most outcomes. However, women reported lower quality of life at discharge and experienced greater treatment benefit at this point compared to men (64). Instead, baseline status emerged as the most consistent predictor of change, with greater improvements observed among patients who started with poorer adherence, lower HRQoL, or higher burden levels. These findings suggest that this kind of intervention, which is multicomponent, high-intensity and patient-centered in transitional care of complex multimorbid patients may be particularly effective in reaching those most in need, regardless of other individual characteristics. These results are to be expected given that scientific evidence suggests that more complex patients benefit more from transitional care interventions (55–57).

### 4.1 Strengths and limitations

The present study has several limitations that should be acknowledged. Firstly, the pre-post design without a control group limits the ability to attribute observed changes—both in primary and secondary outcomes—exclusively to the SPICA intervention, as other external factors may have contributed. In this regard, improvements may partly reflect natural recovery following hospital discharge, as patients often present their lowest scores for quality of life and functional capacity during hospitalization. Secondly, the interpretation of improvements in adherence and other outcomes is limited by methodological constraints. On the one hand, regression to the mean may have influenced the results, as patients with complex conditions and low baseline scores are statistically more likely to show some improvement at follow-up regardless of the intervention. On the other hand, outcomes were assessed only at baseline and one-month post-discharge, which may not adequately capture longer-term patterns or the sustainability of these changes. This short follow-up period may also help explain why improvements were observed in mobility, pain/discomfort, and anxiety/depression, but not in self-care and usual activities, as significant functional recovery in a complex, multimorbid population is unlikely to be detectable within just 1 month. Thirdly, although the recruitment was prematurely halted due to the discontinuation of the program, and the final sample did not reach the initially calculated target of 264 patients, the achieved sample size was sufficient to detect significant changes in the primary outcome of adherence. Nonetheless, the reduced sample may have limited the statistical power for secondary outcomes and subgroup analyses, particularly where effect sizes were smaller, and results from these analyses should therefore be interpreted with caution. Fourthly, all outcomes were based on self-reports, and the lack of blinding may have introduced response and detection bias. Although validated instruments were used, there remains the potential for social desirability bias, particularly in the context of an intensive intervention in which patients may have wished to please the research team. In addition, the increased attention participants received as part of the study (visits, questionnaires, and follow-ups) could, in itself, have promoted better adherence and well-being—a potential Hawthorne effect—regardless of the specific content of the intervention. Fifthly, the study experienced a high rate of refusal among potential participants, which could have influenced the results and introduced selection bias. The authors consider that this high refusal rate may be partly explained by the characteristics of the included patients. Not only are they generally older than the typical study population and less accustomed to participating in this kind of research, but their high clinical complexity places them in a situation of increased clinical and social vulnerability. This may limit their willingness to participate, as they often feel overwhelmed by their own health problems and psychosocial circumstances at the time of recruitment. Finally, as this is evaluative research in health services, it is highly influenced by the healthcare and organizational context and may not be directly extrapolated to other contexts.

Despite these limitations, the study also has notable strengths. A major strength is the comprehensive evaluation of multiple dimensions relevant to patients with complex needs, including adherence, satisfaction, quality of life, and burden of disease and treatment. Additionally, the study employed validated instruments and a mixed analytical strategy, combining pre-post comparisons with multivariate models adjusted for relevant clinical and sociodemographic variables. Another strength lies in its pragmatic approach, reflecting real-world clinical practice and enhancing ecological validity. Furthermore, although the design was not randomized, the significant improvements observed and the robustness of the analyses contribute valuable preliminary evidence for the potential effectiveness in this patient population.

### 4.2 Conclusion

This study suggests an association between a multicomponent, patient-centered transitional care intervention and improvements in adherence, patient satisfaction, and health outcomes in complex multimorbid patients transitioning from hospital to primary care. Nevertheless, further research using more robust methodological designs is needed to confirm and strengthen these findings.

## Data Availability

The datasets presented in this article are not readily available because of concerns regarding patient anonymity. Requests to access the datasets should be directed to the corresponding author.

## Glossary

ADL: Activities of Daily Living
ANCOVA: Analysis of Covariance
DBMA: Disease Burden Morbidity Assessment
EQ-5D-5L: EuroQol 5-Dimension 5-Level
EQ-VAS: EuroQol Visual Analogue Scale
HRQoL: Health-Related Quality of Life
IQR: Interquartile Range
Katz ADL: Katz Index of Independence in Activities of Daily Living
M: Mean
MMAS-4™: Morisky Medication Adherence Scale – 4 item version
*N*: number of patients
*p*: *p*-value
Post: Post-intervention
Pre: Pre-intervention
*R* ^2^: Coefficient of determination
SCS: Canary Islands Health Service
SD: Standard Deviation
SE: Standard error
SPICA: Subprogram for Care Integration and Coordination
TBQ: Treatment Burden Questionnaire
TREND: Transparent Reporting of Evaluations with Nonrandomized Designs
Yrs: Years
*β*: Regression coefficient
