# Correction: A multicomponent, high-intensity, patient-centered care intervention to optimize transitional care coordination for complex multimorbid people: a pre-post design

**DOI:** 10.3389/fmed.2025.1756368

**Published:** 2026-01-06

**Authors:** Tasmania del Pino-Sedeño, Beatriz González de León, Miguel García Hernández, Paula Coronil Olmedo, Yeiza Semiramis Reyes Melián, Vanesa Martínez Hernández, Estefanía García Bautista, Encarnación Barrios Arraez, Silvia Barreto Cruz, Alejandra Abrante-Luis, Miguel Angel García-Bello, Yadira González-Hernández, Juan Antonio López-Rodríguez, José Ramón Vázquez-Díaz

**Affiliations:** 1 Canary Islands Health Research Institute Foundation (FIISC), Tenerife, Spain; 2 Evaluation Unit (SESCS), Canary Islands Health Service (SCS), Tenerife, Spain; 3 Network for Research on Chronicity, Primary Care, and Health Promotion (RICAPPS), Tenerife, Spain; 4 Faculty of Health Sciences, Universidad Europea de Canarias, Tenerife, Spain; 5 Gerencia de Atención Primaria del Área de Salud de Tenerife, Tenerife, Spain; 6 Unidad Docente Multiprofesional de Atención Familiar y Comunitaria “La Laguna Tenerife Norte”, Gerencia de Atención Primaria del Área de Salud de Tenerife, Tenerife, Spain; 7 Research Unit, Primary Health Care Management of Madrid, Madrid Health Service, Madrid, Spain; 8 Medical Specialties and Public Health Department, University Rey Juan Carlos, Madrid, Spain; 9 Primary Care Health Center General Ricardos, Madrid Health Service, Madrid, Spain

**Keywords:** multimorbidity, transitional care, patient-centered care, adherence, quality of life, treatment burden, integrated care, SPICA program

In the published article, the **Funding** statement was incomplete. The original Funding statement read:

“The author(s) declare that financial support was received for the research and/or publication of this article. This work was supported by the Fundación Canaria Instituto de Investigación Sanitaria de Canarias in the Call for Research, Development and Innovation Projects to be carried out in the fields of Hospital Care and Public Health, aimed at meeting the health needs of the population [grant number PIFIISC22/25]. The funder had no role in the design of the study, data collection, analysis, interpretation, or writing of the manuscript.”

The correct **Funding** statement reads:

The author(s) declared that financial support was received for this work and/or its publication. This work was supported by the Fundación Canaria Instituto de Investigación Sanitaria de Canarias in the Call for Research, Development and Innovation Projects to be carried out in the fields of Hospital Care and Public Health, aimed at meeting the health needs of the population [grant number PIFIISC22/25]. The project *also* received a research grant from the Carlos III Institute of Health, Ministry of Economy and Competitiveness (Spain), awarded on the call for the creation of Health Outcomes-Oriented Cooperative Research Networks (RICORS), with reference RD21/0016/0001 and RD21/0016/0013, co-funded by the European Union – NextGenerationEU. The funders had no role in the design of the study, data collection, analysis, interpretation, or writing of the manuscript.

The original version of this article has been updated.

